# Intersectoral actions in decreasing social inequities faced by children and adolescents*

**DOI:** 10.1590/1518-8345.4162.3427

**Published:** 2021-06-28

**Authors:** Larissa Barros de Souza, Francisca Bruna Arruda Aragão, José Henrique da Silva Cunha, Regina Célia Fiorati

**Affiliations:** 1 Universidade de São Paulo, Escola de Enfermagem de Ribeirão Preto, PAHO/WHO Collaborating Centre for Nursing Research Development, Ribeirão Preto, SP, Brazil.; 3 Universidade de São Paulo, Faculdade de Medicina de Ribeirão Preto, Ribeirão Preto, SP, Brazil.

**Keywords:** Intersectoral Collaboration, Socioeconomic Factors, Healthcare Inequality, Child, Adolescent, Vulnerable Populations, Colaboração Intersetorial, Fatores Socioeconômicos, Desigualdade em Saúde, Criança, Adolescente, Populações Vulneráveis, Colaboración Intersectorial, Factores Socioeconómicos, Desigualdad en Salud, Niño, Adolescente, Poblaciones Vulnerables

## Abstract

**Objective:**

to identify the evidence about the repercussion of intersectoral programs / actions / strategies in the reduction of social inequities experienced by children and adolescents in social vulnerability.

**Method:**

integrative review performed in the following databases: National Library of Medicine, Cumulative Index to Nursing and Allied Health Literature, Latin-American and Caribbean Health Sciences Literature, Web of Science, Scopus, and Scientific Electronic Library Online. Primary studies published between 2005 and 2019, written in English, Portuguese, or Spanish, were included. The Rayyan tool was used during selection. The sample was composed of 27 studies, and Ursi was used to extract data. The studies’ methodological quality was verified with the Mixed Methods Appraisal Tool, and descriptive statistics were used.

**Results:**

the main results show that intersectoral actions resulted in improved access to health, improved child nutrition indicators, better mental health care, the adoption of a healthy lifestyle, and improved quality of life.

**Conclusion:**

significant advancements found in the development and lives of children and adolescents are assigned to intersectoral actions. The studies report that different strategies were used in different regions worldwide and contributed to improved children’s and adolescents’ quality of life, supporting new intersectoral policies.

## Abstract (pt)

**Objetivo:**

descrever as evidências sobre a repercussão de programas/ações/estratégias intersetoriais na redução de iniquidades sociais vivenciadas por crianças e adolescentes em vulnerabilidade social.

**Método:**

revisão integrativa realizada nas bases de dados National *Library of Medicine, Cummulative Index to Nursing and Allied Health Literature*, Literatura Latino-Americana e do Caribe em Ciências da Saúde, *Web of Science, Scopus e Scientific Eletronic Library On Line*. Foram considerados estudos primários publicados entre os anos 2005 e 2019, em inglês, português e espanhol, sendo utilizada plataforma Rayyan durante a seleção. A amostra foi composta de 27 estudos. Para extração dos dados foi utilizado instrumento Ursi, a qualidade metodológica dos estudos foi avaliada com o *Mixed Methods Appraisal Tool* e a análise elaborada de forma descritiva.

**Resultados:**

os principais resultados mostram que ações intersetoriais repercutiram em melhorias no acesso à saúde, nos indicadores de nutrição infantil, na qualidade de atendimentos voltados à saúde mental, aumento de hábitos saudáveis e aspectos da qualidade de vida.

**Conclusão:**

avanços significativos na vida e desenvolvimento da população infantojuvenil têm sido atribuídos à intersetorialidade. Os estudos apontaram diferentes estratégias, em diferentes regiões do mundo, cujas repercussões contribuíram para melhorar a qualidade de vida de crianças e adolescentes e subsidiar novas políticas intersetoriais.

## Abstract (es)

**Objetivo:**

describir la evidencia sobre las repercusiones de programas/acciones/estrategias intersectoriales en la reducción de iniquidades sociales experimentadas por niños y adolescentes en vulnerabilidad social.

**Método:**

revisión integradora realizada en las bases de datos *National Library of Medicine, Cummulative Index to Nursing and Allied Health Literature*, Literatura Latino Americana y del Caribe en Ciencias de la Salud, *Web of Science, Scopus y Scientific Eletronic Library On Line*. Fueron considerados estudios primarios publicados entre los años de 2005 y 2019, en inglés, portugués y español, habiendo siendo utilizada la plataforma Rayyan durante la selección. La muestra estuvo compuesta por 27 estudios. Para extracción de los datos fue utilizado el instrumento Ursi; la calidad metodológica de los estudios fue evaluada con el *Mixed Methods Appraisal Tool* y el análisis fue elaborado de forma descriptiva.

**Resultados:**

los principales resultados mostraron que las acciones intersectoriales repercutieron en mejorías en el acceso a la salud, en los indicadores de nutrición infantil, en la calidad de atenciones dirigidas a la salud mental, en aumento de hábitos saludables y aspectos de la calidad de vida.

**Conclusión:**

avances significativos en la vida y desarrollo de la población infantojuvenil han sido atribuidos a la intersectorialidad. Los estudios apuntaron diferentes estrategias, en diferentes regiones del mundo, cuyas repercusiones contribuirán para mejorar la calidad de vida de niños y adolescentes y subvencionar nuevas políticas intersectoriales.

## Introduction

Many children and adolescents in various countries worldwide have little or no access to quality health services or education, good nutrition, or adequate sanitation^(^ 1 ^-^ 3 ^)^. Unequal access to social rights and basic resources required to promote satisfactory development is intrinsically linked to the individuals’ social class, that is, mainly children and adolescents from families experiencing social vulnerability, living in impoverished areas, are affected^(^ 4 ^)^.

According to the Global Strategy for Women’s, Children’s and Adolescents’ Health (2016-2030)^(^ 5 ^)^, one in every three children (200 million worldwide) is unable to reach her/his full physical, cognitive, psychological, and socioemotional potential due to poverty, poor health, and malnutrition, insufficient care, and stimulation, in addition to other risk factors influencing development in early childhood. The impact of poverty on the health and well-being of children and adolescents may affect their participation in occupations (school, leisure, self-care) and relationships, including physical and mental health problems and risk behavior^(^ 6 ^-^ 9 ^)^.

Poverty and inequality increased worldwide, mostly due to the globalization of economies and work restructuring, leading to increased unemployment rates and the breakdown of social ties. Hence, due to persistent social inequality, societies continue to violate the rights of children and adolescents from low-income families, maintaining a context of inequality for this age group^(^ 4 ^,^ 10 ^-^ 11 ^)^.

Previous studies addressing intersectoral actions implemented among socially vulnerable populations report relevant results in decreasing social inequalities, such as improved health services and education, increased income, improved health, empowered vulnerable groups, increased social capital, social participation, and mobilization^(^ 12 ^-^ 16 ^)^.

Intersectoral actions are intended to connect different individuals from various sectors and fields of knowledge to overcome the fragmentation of knowledge and interventions. It represents a new way of facing complex problems^(^ 17 ^-^ 19 ^)^.

The World Health Organization’s Commission of Social Determinants of Health (CSDH-WHO) suggests intersectoral strategies should be adopted to deal with health inequalities considering that most of the problems impacting human health, which are caused by unequal access to quality services and treatments, decent material and psychosocial conditions of life, are social and directly dependent on how society is structured. Therefore, in addition to the health sector, various sectors need to work together to deal with inequalities. Based on the Sustainable Development Goals (SDG), the United Nations considers that eradicating poverty worldwide is an essential condition for development, and intersectoral actions are an essential strategy to be adopted by the various countries^(^ 10 ^,^ 20 ^)^.

The literature presents studies addressing intersectoral actions mainly in adult populations, and there is a gap in terms of studies reporting the results of intersectoral programs directed to children and adolescents. This gap motivated this literature review, the objective was to identify what has been done in different countries in terms of intersectoral strategies to decrease social inequities affecting this population. Hence, an integrative review was conducted to identify the evidence about the repercussions of programs/actions/intersectoral strategies in decreasing social inequities experienced by socially vulnerable children and adolescents.

## Method

This integrative review was based on the following stages: the establishment of the study question (problem identification), search and assessment of primary studies, data analysis, and presentation^(^ 21 ^)^.

The following guiding question was structured using the PICO^(^ 22 ^)^ strategy (Figure 1): “What are the repercussions of intersectoral programs/actions/actions/interventions on decreasing social inequalities experienced by socially vulnerable children and adolescents?”

**Figure 1:** Description of the PICO strategy

Acronym	Definition	Description
P	Patient or problem	Socially vulnerable children and adolescents
I	Intervention	Intersectoral actions
C	Control or comparison	-----------
O	Outcomes	Decreased social inequalities

Primary studies were searched from June to July 2019 in the following databases: MEDLINE/PubMed (via National Library of Medicine), CINAHL (Cumulative Index to Nursing and Allied Health Literature), LILACS (Latin-American and Caribbean Health Sciences Literature), Web of Science, Scopus and SciELO (Scientific Electronic Library Online).

The search strategy included controlled descriptors and key words and Boolean operators: - PubMed, CINAHL, Web of Science, Scopus e SciELO: ((Intersector* OR “cross-sector*” OR “inter-sector*” OR “intersectoral collaboration”) AND (program OR programs OR action OR actions OR strateg* OR policy OR policies OR intervention*) AND (child OR children OR childhood OR adolescen*)); - LILACS: (tw:((Intersetoria$ OR Intersector$ OR “colaboracion intersectorial”))) AND (tw:((programa$ OR ação OR ações OR accion OR acciones OR estrategia$ OR politica$))) AND (tw:((criança$ OR adolescen$ OR nino$))).

Primary studies published between 2005 and 2019, written in English, Portuguese, or Spanish, were selected. This timeframe was chosen because 2005 was when the Commission for Social Determinants of Health was created by the WHO, providing guidelines to implement intersectoral strategies and highlighting the essential role of these strategies in dealing with social inequalities.

Inclusion criteria were: papers reporting the repercussions of intersectoral actions/strategies/programs/interventions and strategies directed to children and/or adolescents experiencing social vulnerability. Exclusion criteria were: papers reporting strategies that had not been implemented or did not report results.

Two reviewers independently selected the studies using the Rayyan^(^ 23 ^)^ tool. The papers were initially selected by their titles and abstracts. The full texts of the studies that met the eligibility criteria and reached a consensus between the reviewers were read to be either included or excluded.

Data were extracted from the primary studies using the Ursi^(^ 24 ^)^ tool, composed of five items: Identification, Host institution, Type of publication, Methodological characteristics, and Assessment of methodological rigor. Three authors independently performed this stage.

Descriptive statistics were used in data analysis. A summary table was organized with the papers’ following information: reference (author and year of publication), study’s objective, study design, sample details, intersectoral action/strategy/program/intervention, and results (concerning decreased social inequities among children and adolescents). The studies’ quality was assessed using the Mixed Methods Appraisal Tool (MMAT). MMAT was developed for reviews including qualitative, quantitative, or mixed methods. It assesses the quality of studies according to five categories: ^(^ 1 ^)^ qualitative studies, ^(^ 2 ^)^ randomized clinical trials, ^(^ 3 ^)^ non-randomized studies, ^(^ 4 ^)^ quantitative descriptive studies, and ^(^ 5 ^)^ mixed methods. There are five quality criteria for each category and scores range from zero (when no criterion is met) to five (all criteria are met)^(^ 25 ^)^.

Because this is a review and does not involve human subjects, there was no need to submit it to the Institutional Review Board. Standards for Quality Improvement Reporting Excellence 2.0 (SQUIRE 2.0) guided all the steps involved in this study’s development.

## Results

**Figure 2:**
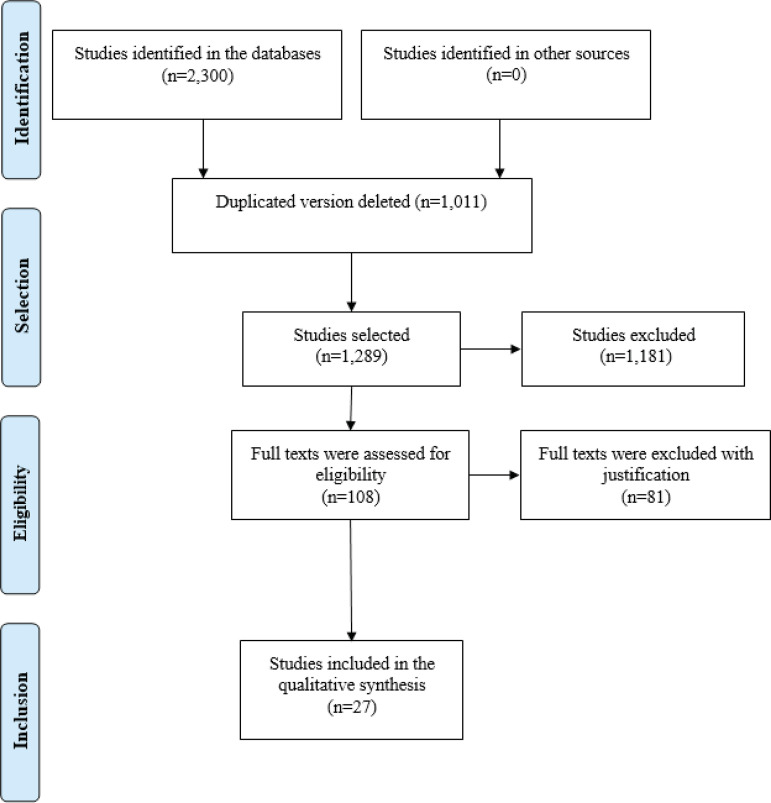
Study Selection Flowchart according to PRISMA^(^ 26 ^)^ guidelines. Ribeirão Preto, SP, Brazil, 2019

A total of 2,300 potentially eligible studies were identified in the databases (Scopus=730, Web of Science=425, PubMed=414, LILACS=336, CINAHL=257, SciELO=138). After importing the studies to the Rayyan platform, 1,011 duplicated versions were excluded. The titles and abstracts of the remaining studies (n=1,289) were read, and another 1,181 records were excluded. The full texts of the remaining papers (n=108) were submitted to inclusion criteria, and 81 were excluded: 28 studies did not address intersectoral actions, 23 emphasized the importance of intersectoral actions but did not report any results, 14 studies reported actions that were not directed to children and adolescents, nine were not primary studies, and seven did not address socially vulnerable populations. Hence, this review is composed of 27 primary studies (Figure 2). Note that no other sources were searched, such as manually seeking the references of the primary studies included in this review or gray literature.

Figure 3 presents the 27 studies characterized according to the author(s), year, country of origin, study design, and assessment of quality according to MMAT. As for the year of publication, the papers included were published from 2008 to 2019, though most papers were published between 2014 and 2019^(^ 26 ^)^, with only one published in 2008. Note that six studies were published in 2016, five in 2019, and four studies were published in 2018, 2017 and 2015, while three studies were published in 2014.

**Figure 3:** Characterization of primary studies according to author(s), year of publication, country of origin, study design, and assessment of quality according to MMAT. Ribeirão Preto, SP, Brazil, 2019

Author	Year	Country of origin	Study design	MMAT *
Appleby, et al.^(^ 27 ^)^	2019	Etiopia	-----------	5/****
Appleby, et al.^(^ 28 ^)^	2019	Nueva Zelandia	Abordaje cualitativo	1/*****
Barrett, et al.^(^ 29 ^)^	2016	EUA	-----------	3/*****
Chandra-Mouli, et al.^(^ 30 ^)^	2018	India	Cualitativo y cuantitativo	5/****
Fabbiani, et al^(^ 31 ^)^	2016	Uruguay	Relato de Experiencia - cualitativo	1/*****
Fabelo-Roche, et al.^(^ 32 ^)^	2016	Cuba	Cualitativo	1/*****
Ferrugem, et al.^(^ 17 ^)^	2015	Brasil	Relato de experiencia - cualitativo	1/*****
Gimenez, et al.^(^ 33 ^)^	2014	Brasil	Cualitativo	1/*****
Jones, et al.^(^ 34 ^)^	2019	Australia	Descriptivo	1/*****
Laurin, et al.^(^ 35 ^)^	2015	Canadá	Estudio de casos múltiples - interpretativo	1/*****
Leite, et al.^(^ 36 ^)^	2015	Brasil	Relato de experiencia - cualitativo	1/*****
Melo, et al.^(^ 37 ^)^	2016	Brasil	Estudio de caso con abordaje cualitativo	1/*****
Milman, et al.^(^ 38 ^)^	2018	Chile	Estudio de caso	1/*****
Mongiovi, et al.^(^ 39 ^)^	2018	Brasil	Relato de experiencia - cualitativo	1/*****
Monteiro, et al.^(^ 40 ^)^	2015	Brasil	Investigación acción con abordaje cualitativa	1/*****
Moyano, et al.^(^ 41 ^)^	2018	Argentina	Investigación acción con evaluación cualitativa	1/*****
Nunes, et al.^(^ 42 ^)^	2016	Brasil	Exploratorio descriptivo con abordaje cualitativo	1/*****
O'Malley, et al.^(^ 43 ^)^	2017	EUA	Estudio de caso	1/*****
Obach, et al.^(^ 44 ^)^	2019	Chile	Cualitativo etnográfico	1/*****
Obach, et al.^(^ 45 ^)^	2017	Chile	Cualitativo etnográfico	1/*****
Pappas, et al.^(^ 46 ^)^	2008	Paquistán	-----------	5/*****
Reader, et al.^(^ 47 ^)^	2017	Nueva York	-----------	1/*****
Shan, et al.^(^ 48 ^)^	2014	Canadá	Mixto	5/****
Tãno, et al.^(^ 49 ^)^	2019	Brasil	Exploratorio y de levantamiento, con triangulación de métodos	5/*****
Tkac, et al.^(^ 50 ^)^	2017	Brasil	Longitudinal, del tipo experimental	3/****
Torricelli, et al.^(^ 51 ^)^	2014	Argentina	Transversal analítico descriptivo, cualitativo y cuantitativo	5/*****
Woodland, et al.^(^ 52 ^)^	2016	Australia	Abordaje de métodos mixtos	5/*****

* Los números y asteriscos se refieren, respectivamente, a la categoría del diseño de los estudios y a la clasificación de calidad de los estudios, de acuerdo con el MMAT

According to the geographical distribution, the studies were published in different regions: South America, North America, Central America, Africa, Asia, and Oceania. Brazil was the country with the highest number of studies, nine(17,33,36-37,39-40,42,49-50), followed by Chile(38,44-45) and the United States(29,43,47) with three studies each, then Canada^(^ 35 ^,^ 48 ^)^, Argentina^(^ 41 ^,^ 51 ^)^, and Australia^(^ 34 ^,^ 52 ^)^, with two studies each. The remaining countries, Ethiopia^(^ 27 ^)^, New Zealand^(^ 28 ^)^, India^(^ 30 ^)^, Uruguay^(^ 31 ^)^, Cuba^(^ 32 ^)^, and Pakistan^(^ 46 ^)^ presented one study each.

The nomenclature used by the authors of the studies included here was maintained. Most were qualitative studies, four of which were experience reports^(^ 17 ^,^ 31 ^,^ 36 ^,^ 39 ^)^, four were case studies^(^ 35 ^,^ 37 ^-^ 38 ^,^ 43 ^)^ (one was a multiple case study^(^ 35 ^)^), three were only reported as qualitative studies^(^ 28 ^,^ 32 ^-^ 33 ^)^, two were ethnographic studies^(^ 44 ^-^ 45 ^)^, two were action research^(^ 40 ^-^ 41 ^)^, one was an exploratory, descriptive study^(^ 42 ^)^, and one was a descriptive study^(^ 34 ^)^. Other two studies presented a mixed approach^(^ 48 ^,^ 52 ^)^, one was an exploratory study with the triangulation of methods^(^ 49 ^)^, one was a descriptive cross-sectional analysis with a quantitative-qualitative approach^(^ 51 ^)^, one was an experimental longitudinal study^(^ 50 ^)^, and there were four studies in which the study design was not clearly reported^(^ 27 ^,^ 29 ^,^ 46 ^-^ 47 ^)^.

Regarding the implementation of MMAT, the studies were classified regarding their category and the methodological quality of each group was analyzed separately. The numbers from 1 to 5 identify each category of study design according to the tool used. Hence, 18 studies^(^ 17 ^,^ 28 ^,^ 31 ^-^ 45 ^,^ 47 ^)^ fit a qualitative approach^(^ 1 ^)^, seven^(^ 27 ^,^ 30 ^,^ 46 ^,^ 48 ^-^ 49 ^,^ 51 ^-^ 52 ^)^ used mixed methods^(^ 5 ^)^ and two studies^(^ 29 ^,^ 50 ^)^ presented a non-randomized quantitative approach^(^ 3 ^)^. Twenty-three out of the 27 papers were classified as high-quality studies (*****), among which all the qualitative studies, four studies with mixed methods, and one non-randomized quantitative study, as they met all the five criteria. The remaining papers^(^ 27 ^,^ 30 ^,^ 48 ^,^ 50 ^)^ also presented high methodological quality, though met four of the five criteria (****). Thus, there were no low-quality studies.

Data concerning the intersectoral actions identified in the studies and their respective results in decreasing social inequalities experienced by children and adolescents are presented in Figure 4.

As for the sectors involved in the actions reported, all the studies mention the health sector, while education is mentioned in 23 studies^(^ 17 ^,^ 27 ^,^ 29 ^,^ 31 ^-^ 42 ^,^ 44 ^-^ 50 ^,^ 52 ^)^. There were also actions concerning mental health^(^ 28 ^-^ 29 ^,^ 42 ^-^ 43 ^,^ 49 ^,^ 51 ^)^, feeding^(^ 27 ^,^ 37 ^,^ 46 ^-^ 47 ^,^ 50 ^)^ and more specific issues, such as youth justice^(^ 28 ^-^ 29 ^,^ 36 ^)^, and sexual education^(^ 44 ^-^ 45 ^)^. Actions in the remaining papers addressed broader and more comprehensive topics such as health counseling^(^ 31 ^)^, quality of life^(^ 33 ^,^ 41 ^)^, full development^(^ 38 ^)^, violence prevention^(^ 40 ^)^, and decreased (social, health and education) inequities^(^ 34 ^)^.

**Figure 4:** Characterization of primary studies according to author(s), year of publication, intersectoral action, and results (concerning decreased social inequalities among children and adolescents). Ribeirão Preto, SP, Brazil, 2019

Reference (author/year)	Action/Strategy/Policy/Intersectoral intervention	Results (concerning decreased social inequities among children and adolescents)
Appleby, et al. (2019)^(^ 27 ^)^	Enhanced School Health Initiative - school health and feeding program in Ethiopia.	- Improvement in the main health and child nutrition indicators included decrease prevalence and intensity of parasite infection - Improvement in hygiene behavior and sanitation among school-aged children
Appleby, et al. (2019)^(^ 28 ^)^	Information sharing strategy on the mental health needs of young people living in juvenile justice homes in New Zealand - involving mental health and justice.	- Information sharing - Appropriate information regarding mental health - Support to workers so they could provided better service to the young individuals
Barrett, et al. (2016)^(^ 29 ^)^	Safety Net Collaborative, collaborative partnership between the police, mental health care providers, schools and human services, to prevent the incarceration of young individuals and improve access to mental health services in Cambridge, Massachusetts, USA.	- Community detentions decreased by more than 50% - The hiring of mental health services raised the average of outpatient medical consultations per year
Chandra-Mouli, et al. (2018)^(^ 30 ^)^	Multi-sector intervention implemented at a district level to deal with child marriage in Rajasthan, India.	- Cascade effect to encourage combined actions at the block and village level - Non-governmental organization committed to provide support - Design and implementation specific to the context and a flexible and responsive approach - enlist leaderships of key government officials in accordance with the duties described in the 2006 Child Marriage Prohibition Act
Fabbiani, et al. (2016)^(^ 31 ^)^	Integral Health Guidance and Hearing Spaces Project in Educational Centers, a strategy of sharing centers and health integral counseling in educational centers in Montevideo, Uruguay - involving social, health and educational services.	- Students occupy the space, participate spontaneously and value the proposal - Most consultations are resolved immediately, offering care and opportune orientations - Distress and discomfort are decreased by taking care of old problems that are detected for the first time within the program - Response to more complex situations is coordinated with the educational community, family and networks
Fabelo-Roche, et al. (2016)^(^ 32 ^)^	Workshops with participatory and dynamic techniques to decrease alcohol consumption among Cuban adolescents - collaboration among academic and educational sectors and the business sector.	- No additional student initiated alcohol consumption during the intervention - Indicators improved suggesting a change in healthy cultural and recreational activities with vocational aspirations being included in plans of life - Risk perception concerning alcohol and drug consumption increased considerably - Negative attitudes toward alcohol increased
Ferrugem, et al. (2015)^(^ 17 ^)^	Bonde do Cine Project: discussing cinema, producing heath in Porto Alegre, RS, Brazil - involving health, education, and culture.	- Collective interventions, with exchange of experiences, social participation, horizontal dialogue promoted a joint development of knowledge, strengthening individuals and encouraging a critical reflection upon the different topics related to the adolescents' routine - Important contribution to the educational process of students and teachers
Gimenez, et al. (2014)^(^ 33 ^)^	Programa Saúde na Escola (PSE) [Health at School Program] in Marília, SP, Brazil - intersectoral policy of the Ministry of Health and Ministry of Education to improve the quality of life of children, adolescents, and adults by proposing policies and actions implemented by the health and education sectors within schools.	- Considerable increase in the demand of serological tests among individuals younger than 18 years old, as well as the distribution of condoms in Primary Health Care Centers - A co-responsibility process expanded the capacity of each sector/area to analyze and transform practice from the perspective of other sectors/areas, leading to more effective results - Greater visibility to the multi-causal determinants of the health-disease continuum with the participation all sectors in action intending to break the fragmentation of healthcare when facing the various problems presented by these groups
Jones, et al. (2019)^(^ 34 ^)^	Strategy intended to address inequalities in the health, education, and social spheres among rural children and adolescents from Australia - collaboration among the local health district, schools, and a university rural health department.	- Improved relationships, resources, and workforce - Promoted health coordination and integration
Laurin, et al. (2015)^(^ 35 ^)^	Survey on school readiness of children in districts in Montreal, Canada - partnership among health care networks, education, daycare services, community and charity organizations, and the Ministry of Immigration.	- Greater visibility of child development and its importance, impacting those involved in early childhood, who felt prepared to draw the attention of other bodies to the situation - Intersectoral committees implemented in all the territories to organize and follow-up the local government resulted in expanding and consolidating partner networks - Intersectoral actions ensure the support of a larger range of services, encompassing various spheres of child development, with greater visibility and access within the community
Leite, et al. (2015)^(^ 36 ^)^	State Operational Plan of Integral Health Care provided to Adolescents Deprived of Freedom and its effective implementation in Acre, Brazil - cooperation with the State Public Ministry, Socioeducational Institute, State Health Department, State Education Department, and Municipal Health Departments, and Rio Branco Social Service.	- Expanded the involvement of actors from the adolescent care and protection network - Enhanced co-responsibility of the various services in the health care network
Melo, et al. (2016)^(^ 37 ^)^	School Feeding Program in Itabira, PE, Brazil - involving education, health and social sectors.	- Results concerning the organizational and sociopolitical contexts: program institutionalization, efficient use of financial resources, municipal management, high community participation, and the use of local resources to favor the program
Milman, et al. (2018)^(^ 38 ^)^	Chile Cresce Contigo Program [Chile grows with you program] to help all children to reach their development potential, regardless of their socioeconomic conditions, supporting children and families - involved health, social protection, and education sectors.	- Positive effects on child development - The more the families use the program benefits and the longer the subsystem operates in the community, the greater the positive effects
Mongiovi, et al. (2018)^(^ 39 ^)^	Educational intervention to cope with homophobia implemented among adolescents in a high school in Recife, PE, Brazil - involving health and education.	- Establishing opportunities for participation and dialogue to cope with homophobia within school - Promoting health and integral and civil education to adolescents to deal with social vulnerability and violence
Monteiro, et al. (2015)^(^ 40 ^)^	Culture Circles - dynamic learning opportunities in which knowledge concerning strategies to prevent violence is collective developed in Recife, PE, Brazil - health education intervention addressing adolescents.	- Educational action promoted a critical sociopolitical and cultural stance among adolescents in the face of vulnerability to violence, including the guarantee of human rights, justice, and combat inequities - Changes in social relationships, fighting discrimination and intolerance - Expanded access and reorientation of health services through intersectoral public policies
Moyano, et al. (2018)^(^ 41 ^)^	Project based on agro-ecological systems to improve some dimensions of quality of life and of the school environment in Argentina - it involves education, health, social and environmental sectors.	- Teachers reported the positive impact of the project on the adolescents' school level - Positive contributions on the adolescents' quality of life aspects, both objective and subjective aspects, feasible to be implemented in the school environment through intersectoral actions
Nunes, et al. (2016)^(^ 42 ^)^	Actions directed to the mental health of children and adolescents São Lourenço do Sul, RS, Brazil - cooperation among network services (health, education, social services, and justice).	- Greater problem-solving capacity to meet the needs of children and adolescents - The various sectors involved, regardless of the sphere they represent, are committed with the integral protection of these individuals - Efficient strategies in the continuity of care, contributing to enrich new possibilities of interventions
O'Malley, et al. (2017)^(^ 43 ^)^	Innovative collaboration to deal with toxic stress among children growing up in poverty in Kansas City, USA - between a community center, Breakthrough Operation and a tertiary child hospital.	- Data sharing agreements allow clinicians to know what care actions were provided to the children and what other care actions are needed - Children started receiving timely and non-redundant care - Cooperation and collaboration are apparent at school, clinic, administration and philanthropic departments at the Children's Mercy Hospital (CMH) and Breakthrough Operation
Obach, et al. (2019)^(^ 44 ^)^	Strategies to address sexual and reproductive health among adolescents, to prevent adolescent pregnancy and explore the perceptions of adolescents and health workers regarding the implementations of these strategies in Chile - involved the health and education sectors.	- Facilitated the access of adolescents to mental and reproductive health - Enabled sexual and reproductive health to be seen as an integral dimension of life and reinforced the holistic notion of health - Encouraged the health sector to connect with the community and share responsibility for health care - Facilitated the exercise of rights and improved the well-being of adolescents within the community, contributing to a healthier community as pregnancy-associated risks decrease as well as the reproduction of poverty and gender inequalities
Obach, et al. (2017)^(^ 45 ^)^	Friendly Services Program, a strategy to question the adolescents' perceptions regarding sexual education in the Metropolitan region of Chile - involved the health and education sectors.	- Cooperative and coordinated work among sectors - Improved response to the adolescents' needs concerning sexual information and education
Pappas, et al. (2008)^(^ 46 ^)^	Tawana Pakistan Project (TPP), school eating program - meals were provided in elementary schools by the Pakistan government - involved health and education sectors and the community.	- Waste decreased by half and school enrollment increased by 40% - Malnutrition decreased and the communities' knowledge regarding diet improved - Three nutritional status measures improved: acute malnutrition decreased by 45%; the number of underweight girls decreased by 21.7%; short stature, a measure of chronic malnutrition, decreased by 6% - Various improvements were found in the schools included in the project: the number of teachers increased, school discipline improved, the number of schools increased with improved infrastructure including latrines and water supply, and improved hygiene measures adopted by the schools' kitchens
Reader, et al. (2017)^(^ 47 ^)^	Wellness in the Schools Internship Program to fight obesity among children of public schools in New York, USA - partnership between a non-profit organization and a urban community college.	- Repeated exposure to healthy foods changed the behavior of some school-aged children toward healthy diet, showing positive attitudes - Some students became interested in trying new foods - College students were positive role models due to their age, ethnicity, and life experience they shared with the children
Shan, et al. (2014)^(^ 48 ^)^	KidsFirst, an early childhood intervention program directed to vulnerable families in target-areas in Saskatchewan - Canada including efficient practices to improve social capital and social cohesion at the community and institutional levels.	- The community social fabric was strengthened, uniting the community, cultivating community social capital and improving the institutional environments and services - Improved the awareness of the community regarding the health of children - Gained support from different organizations that assisted fundraising, donating medications, providing free services, and disseminating health information - It played a central role in connecting parents with health and other services
Tãno, et al. (2019)^(^ 49 ^)^	Situations that demand care among children and adolescents cared for by Psychosocial Care Centers (CAPsij) located in the Southeast of Brazil - the main sectors involved are health, education and social service.	- Support networks were created for the services' users and professionals - Communication and exchange of knowledge, thoughts, and experiences were expanded, leading to a sense of partnership and contact that relieves work overload and sustains the duration of interventions - The perception of educators improved regarding the mental health of children and adolescents in psychological distress
Tkac, et al. (2017)^(^ 50 ^)^	Program intended to promote the health of school-aged children through physical activity and healthy diet in Curitiba, PR, Brazil - support was provided by the city education and health departments, school management, and research groups of public and private universities.	- Long-term interventions promoting positive and significant changes in the school health indicators - Behavioral changes through empowering students, managers and parents
Torricelli, et al. (2014)^(^ 51 ^)^	Community-based program directed to children and adolescents with mental health problems in Buenos Aires, Argentina.	- A larger number of children and adolescents with significant psychological distress and psychosocial vulnerability, improving accessibility and general conditions - Designed and implemented comprehensive and territorialized responses, ensuring effective intersectoral responses, resulting in positive assessment
Woodland, et al. (2016)^(^ 52 ^)^	Optimizing Health and Learning Program intended to a transferable and sustainable care mode to improve health outcomes and learning among refugees and other young migrants in Sidney, Australia.	- Detection of health conditions with the potential to cause impact on the health and learning of students was improved - Recently arrived students and their families are connected to primary health care services - Primary health care and specialized services were coordinated

The main results show various advancements concerning decreased social inequities among children and adolescents experiencing social vulnerability, such as improved access to health services, child nutrition indicators, information, quality, and number of consultations directed to mental health, healthy lifestyle, and improved quality of life^(^ 27 ^-^ 29 ^,^ 31 ^-^ 33 ^,^ 41 ^,^ 51 ^)^.

Other advancements show interventions that contributed to the educational process of children and adolescents, increased school enrollment, increased indicators suggesting changes toward cultural and healthy recreational activities, vocational aspirations were included in life plans, joint construction of knowledge, critical reflection, and empowerment, creating opportunities for this population to participate and dialogue within the school environment to cope with homophobia, discrimination, and intolerance, in addition to improving the infrastructure of schools^(^ 17 ^,^ 39 ^-^ 41 ^,^ 46 ^-^ 47 ^,^ 50 ^)^.

Other actions and significant results include support to workers providing care to children and adolescents intended to improve services; hiring a larger number of workers; recognizing the importance of co-responsibility and sharing of information; more flexible and responsive approaches; promoting the coordination and integration of care; results concerning organizational and sociopolitical contexts such as the efficient use of financial resources and greater community participation; increased visibility and the importance of child development; strengthening the community social fabric, cultivating social capital, and improving institutional environments and services; more frequent partnerships and contact by expanding communication and exchanging knowledge, alleviating work overload and sustaining the duration, quality and effectiveness of interventions; creating and strengthening support networks and supporting the services’ users and workers^(^ 17 ^,^ 28 ^-^ 31 ^,^ 33 ^-^ 36 ^,^ 43 ^-^ 46 ^,^ 48 ^-^ 49 ^,^ 52 ^)^.

## Discussion

This review’s objective was to identify the repercussions of intersectoral actions directed to children and adolescents in terms of coping with social inequalities. In this sense, the studies addressed here presented important advancements in decreasing social inequities.

The actions and results concerning clear and direct data mainly reflect on the health and education of children and adolescents^(^ 27 ^-^ 28 ^,^ 31 ^-^ 33 ^,^ 39 ^,^ 43 ^-^ 47 ^,^ 51 ^-^ 52 ^)^. At the same time, the results of indirect actions are also reported, showing a considerable impact on the children and young population, such as improved services, on the actions of the professionals working with this population, new partnerships and support, the construction of networks, support to families, increased access to services and information^(^ 17 ^,^ 28 ^,^ 30 ^-^ 31 ^,^ 35 ^-^ 36 ^,^ 40 ^-^ 49 ^)^.

As for the sectors involved in the actions reported, the health sector is reported by all the studies. This is an important sector considering that a healthy society tends to increase its productivity, and as a consequence, there is increased economic returns and greater participation in the job market, expanding the possibilities of more inclusive and sustainable development^(^ 53 ^)^. However, in order for the population to achieve improved health and social well-being, actions implemented in the health sector alone do not suffice. Leadership is needed to encourage intersectoral actions aimed to decrease inequalities^(^ 13 ^)^.

After the health sector, the education sector was the most frequently reported in intersectoral actions, and partnerships established between health and education are evident^(^ 17 ^,^ 27 ^,^ 31 ^,^ 33 ^-^ 34 ^,^ 39 ^,^ 44 ^-^ 46 ^,^ 50 ^,^ 52 ^)^ mostly in South American countries (Brazil, Chile and Uruguay). The school system is an excellent means to implement interventions intended to improve health conditions that most frequently affect school-aged children, improving participation, and learning^(^ 27 ^)^. Health and feeding school programs are the strategies most frequently used in low- and moderate-income countries to provide health education and promote behavioral changes in this population^(^ 54 ^-^ 55 ^)^.

There were feeding programs focusing on the specific problems of each country: one Ethiopian study^(^ 27 ^)^ implemented a health and feeding school program and obtained improved hygiene and sanitation behavior among children, decreasing the prevalence and intensity of parasite infection. A school food program in Pakistan^(^ 46 ^)^ implemented in 4,035 elementary schools decreased acute malnutrition by 45%, underweight drop by 21.7%, and short stature by 6%. In New York^(^ 47 ^)^ a program intended to fight child obesity identified a change of behavior among children toward healthy eating, with positive attitudes and interest in new foods. In Brazil^(^ 50 ^)^, an action intended to promote the health of school-aged children by promoting physical activity and a healthy diet improved school health indicators and behavioral change not only of students but also of workers and parents.

The authors note that school health and feeding programs are among the main services intervening in health conditions that tend to affect school-aged children. The infrastructure provided by schools facilitates the implementation of health programs with reduced costs. Therefore, schools provide health education, improving the access of marginalized families to health care, and promoting behaviors that reflect in improved school enrollment and attendance, and decrease gender differences^(^ 54 ^-^ 55 ^)^.

Still, regarding health education, interventions implemented in schools with the active participation of children and with partnerships established with organizations from other sectors ensure consistency and sustainability of initiatives^(^ 56 ^-^ 57 ^)^.

Among the studies selected, the mental health of children and adolescents is addressed in terms of improved access^(^ 29 ^)^, an increased average number of outpatient consultations/year, and decreased imprisonment of young individuals by more than 50% in a city in the United States; decreased chronic stress among poor children^(^ 43 ^)^, through cooperation and collaboration among sectors, also in the United States; care provided to children and adolescents by a Psychosocial Care Center (CAPSij)^(^ 49 ^)^ in Brazil; and information sharing strategy implemented in youth justice residences (for individuals aged between 12 and 17 who represent a risk to themselves or other people)^(^ 28 ^)^ in New Zealand.

Other studies also report that network actions are more effective and powerful, being a priority in psychosocial care provided to children and adolescents. In this sense, the repercussions of intersectoral actions as a strategy of intervention and management identified in this review are in line with what the authors had already pointed out, showing the structuring of a shared commitment with decreased social inequities and other hardships faced by children and adolescents^(^ 58 ^-^ 60 ^)^. When intersectoral actions are prioritized in mental health services, they enable specific care is provided to this population and contribute to a broader view of psychological distress, breaking away from the biomedical, reductionist, and mechanicist notion^(^ 61 ^-^ 63 ^)^.

The actions directed to young individuals and their relationship with justice seek to work with mental health from a preventive perspective to prevent the incarceration of this population^(^ 29 ^)^, such as promoting the health of those in youth justice residences^(^ 28 ^)^. These are mostly young individuals who had to deal with poverty, social deprivation and were exposed to violence from a very young age, situations that evidence their social vulnerability and the inequities to which they are subject^(^ 28 ^)^. The results show the importance of enabling this population to have access to services. Even though this population is considered to be at risk, there is restricted access to health services before these individuals enter the justice system; few actions are intended to prevent young people from committing crimes^(^ 29 ^)^.

The studies also show that intersectoral actions, even though being a recent and seldom-used strategy to manage programs and public policies in complex situations, have been increasingly adopted to deal with violence and the abusive use of drugs^(^ 13 ^)^. In this sense, there are studies whose actions address more specific issues, which produce profound inequities and which address youths with problems with the law; actions addressing sexual and reproductive education of adolescents^(^ 44 ^-^ 45 ^)^, intending to provide information on how to prevent adolescent pregnancy; actions to cope with homophobia^(^ 39 ^)^, addressing gender, sexual diversity, and human rights; decreased consumption of alcohol among adolescents^(^ 32 ^)^; actions to deal with child marriage^(^ 30 ^)^ in India; to increase social capital and social cohesion^(^ 48 ^)^; and to provide care to refugee children^(^ 52 ^)^ in Australia.

Even though intersectoral actions are considered essential to obtain good results with the implementation of policies, the studies reveal that administrative and managerial difficulties need to be overcome. The problems refer to difficulties in breaking with a sectorial logic that prevents cooperation, distribution of responsibility, and operational actions. Additionally, difficulties related to governments, which promote the centralization of power and deliberative capacity for intersectoral forums, obstacles imposed on the civil society, preventing it from organizing cohesively to claim rights in the face of political power, are important factors^(^ 64 ^-^ 65 ^)^.

Another difficulty in implementing intersectoral projects is the managers’ insufficient technical preparation, while creating a collaborative culture in managerial and administrative relations, together with technical training for intersectoral management, is essential^(^ 13 ^)^.

Even though advancements were verified in many countries, social inequities are predominant factors determining inequalities in the health field and imposing obstacles to establishing equality. Policies in emerging countries addressing the social determinants of health and intended to decrease inequalities are fragmented and show a lack of cooperation in the implementation, management, and inspection of actions. Local governments show important differences in the rhythm and establishment of priorities with which policies are implemented, creating gaps among the same country’s regions. As for Latin American countries, there are problems related to intense cultural, ethnic, poverty and gender issues that need to be addressed for intersectoral projects directed to the production of health equity to be implemented(14-15,66).

The studies included in this review were assessed with MMAT and presented good methodological quality; all studies met four to five of the criteria proposed for each study design. All the qualitative studies were considered to be of high quality as they met all the five criteria. As for the studies that met four criteria, the non-randomized quantitative study presented a failure concerning confounding factors, which were not clearly reported. The most frequent limitation of the mixed studies refers to not properly describing the procedures, which hindered the assessment of the methods used.

This review’s limitations refer to the timeframe and restriction of languages, the non-inclusion of gray literature, and the fact only primary studies were selected. Additionally, the descriptive analysis of data from studies using different methodological approaches may lead to bias when presenting the results.

Nonetheless, this review represents an initial step toward a more in-depth analysis of this topic. According to the UN and WHO’s goals to end global poverty by 2050, by promoting global equity in health, multi-sector, intersectoral, and cross-national actions will be increasingly needed. Therefore, further research and additional evidence are needed to show that intersectoral policies and/or combined with social participation can impact the social determinants of health and decrease social and health inequities.

## Conclusion

The studies included in this review report significant advancements through intersectoral actions, which have helped and increased the potential to achieve more equitable societies.

Reflecting upon this review’s question, the analysis shows that intersectoral strategies produced positive results concerning health, educational level, and quality of life of children and adolescents in the countries and regions in which these experiences were implemented. Additionally, positive results were found for the communities in which these children and adolescents live with an increase in the community’s social capital.

Positive results were found for professionals working with this population, increasing their qualification and quality of the services provided, obtaining more information to implement new intervention projects, and supporting the establishment of public policies.
